# Sequence-Based Typing of *Leptospira:* Epidemiology in the Genomic Era

**DOI:** 10.1371/journal.pntd.0000120

**Published:** 2007-11-07

**Authors:** Paul N. Levett

**Affiliations:** Saskatchewan Disease Control Laboratory, Saskatchewan Health, Regina, Canada; Oswaldo Cruz Foundation, Brazil

## Background

Leptospirosis is a zoonotic disease of global distribution, maintained in nature by chronic renal infection of carrier animals. Infected animals excrete the organisms in their urine and contaminate the environment. Human infection is acquired directly via exposure to the organism in tissues or body fluids, but most often occurs indirectly via exposure to the organisms in the environment. Thus human exposure usually occurs remotely from the reservoir or source animal.

The incidence of leptospirosis is very strongly associated with rainfall. Excess rainfall events that cause massive flooding are associated with the potential for huge outbreaks, particularly in densely populated regions of the tropical world. In such situations it is difficult if not impossible to identify the reservoir(s) of the organisms that cause human cases.

Historically, leptospires were classified into two species, *Leptospira interrogans* and *L. biflexa*, which comprised pathogenic and nonpathogenic strains, respectively. Within these two species, several hundred serovars were recognised [1]. Identification of isolates to serovar level is an essential step to understanding the epidemiology of the disease in both humans and animals in any geographic region. However, serovar identification remains a relatively blunt tool with which to investigate fine details of epidemiology.

Genetic variation observed within the genus *Leptospira* [2],[3] led to the replacement of the historical classification by one based on DNA relatedness [4],[5]. In this system 14 species are currently recognised and at least another six species are in the process of being described. The development of a molecular classification provided the basis for understanding the genetically distinct subtypes of serovar Hardjo. Two genotypes of serovar Hardjo, with distinct biological characteristics and geographical distribution, named Hardjoprajitno and Hardjobovis [6]–[8] are classified within *L. interrogans* and *L. borgpetersenii,* respectively. Evidence of horizontal gene transfer within the genus, mediated by insertion sequences, is widespread [9]–[12].

A wide range of methods based upon restriction endonuclease digests, random amplification, or hybridization have been applied to typing of leptospiral isolates, with moderate success. The availability of increasing numbers of genome sequences has facilitated the application of sequence-based approaches that can yield much deeper information about relationships between strains [13]–[15].

## A New MLST Study

In recent years Thailand has undergone a significant outbreak of leptospirosis [16],[17]. From 1996 to 2000, reported cases increased approximately 40-fold to a peak of 14,000 cases per year [17]. In the absence of climatic or behavioural changes to account for the outbreak, hypotheses for this relatively sudden, explosive increase in incidence include the emergence of a clone of *Leptospira* better equipped for survival in a maintenance host or in the environment, or one of greater virulence. Serological identification of isolates indicated that many were of *L. interrogans* serovar Autumnalis. The recently published paper by Thaipadungpanit and colleagues in *PLoS Neglected Tropical Diseases* [18] brought the discriminatory power of sequence-based typing to bear on the epidemiology of this rapidly emerging disease.

In this study, a multilocus sequence typing (MLST) approach was developed for application to isolates of *Leptospira* from humans and from reservoir host animals. From among 101 human isolates from northeast Thailand, 12 sequence types were identified. A single sequence type (ST34) accounted for 76% of isolates. During the peak of the outbreak in 2000/2001, ST34 accounted for 85% of all isolates. ST34 was also predominant among isolates from other regions of Thailand during the same time period.

Moreover, seven isolates from bandicoot rats (*Bandicota* spp.) were also shown to be ST34, while a single isolate from *Rattus rattus* was of a different sequence type. All isolates of ST34 were identified as *L. interrogans* serovar Autumnalis, but not all Autumnalis isolates were of ST34. In contrast, a collection of 73 reference strains yielded 59 sequence types, emphasising the limited diversity of the Thailand outbreak isolates.

## Implications

The association of a single clone with a large outbreak of leptospirosis leads to several hypotheses, which are discussed eloquently by Thaipadungpanit et al. [18]. Further work will enhance our understanding of why clones such as ST34 expand and become predominant in outbreaks.

Despite the advances offered by application of MLST to leptospiral epidemiology, there are limitations. The authors note the absence of isolates that pre-date the onset of the outbreak, and were thus unable to show conclusively that the outbreak resulted from the expansion of the ST34 clone. In addition, relatively few isolates from animals were available for study. However, the distribution of the clone in Thailand, its decline in relative frequency as the outbreak waned, and its absence from the collection of reference strains and other isolates, are strongly supportive of their conclusion that the ST34 clone was responsible for the outbreak.

The existence of other localised clones has been suggested previously [19],[20]. While a clone might be predicted to become predominant in an isolated island environment, the question of how and why a clone may have spread among bandicoot rats in a relatively large country such as Thailand needs to be answered. On a global scale, might all isolates of *L. interrogans* serovars Copenhageni and Icterohaemorrhagiae from *Rattus norvegicus* be clonal? Prospective studies could be designed to determine the effect on clonal circulation of public health interventions to reduce rodent numbers.

The MLST developed by Thaipadungpanit and coworkers [18] generated sequence data from isolates of *L. interrogans* and *L. kirschneri*, two closely related species of pathogenic leptospires that account for the majority of disease cases, but not from *L. borgpetersenii*. Another recently published MLST using different targets yielded data for six species of pathogenic leptospires [13]. It would be advantageous for any *Leptospira* MLST to be applicable to all leptospires, but at least to all pathogenic species. Further studies will be necessary to determine the combination of targets that is most widely applicable.

One of the major advantages of MLST is the ability to perform evolutionary analyses on data widely spaced in time and geography. The current data illustrate the value of this tool for investigating the local epidemiology of an outbreak. Wider application of this approach to historical and contemporary collections of isolates from around the globe will give us greater insight into the evolutionary biology of this important pathogen.
